# Resource Utilization and Environmental and Spatio-Temporal Overlap of a Hilltopping Lycaenid Butterfly Community in the Colombian Andes

**DOI:** 10.1673/031.009.1601

**Published:** 2009-05-08

**Authors:** Carlos Prieto, Hans W. Dahners

**Affiliations:** ^1^ Centro Iberoamericano de la Biodiversidad (CIBIO), Universidad de Alicante, España; ^2^ Departamento de Ciencias Fisiológicas, Universidad del Valle, Cali, Colombia

**Keywords:** Lycaenidae, theclinae, vertical stratification, niche segregation, Valle del Cauca, Aguacatal River basin

## Abstract

Coexistence by a great number of species could reflect niche segregation at several resource axes. Differences in the use of a hilltop as mating site for a Eumaeini (Lycaenidae) community were measured to test whether niche segregation exists within this group. Specimens were collected throughout 21 samplings between July-October of 2004 and July-October of 2005. Two environmental variables and three temporal-spacial variables were analyzed utilizing null models with three randomization algorithms. Significant differences were found among the species with respect to utilization of vertical space, horizontal space, temporary distribution and environmental temperature. The species did not show significant differences with respect to light intensity. For all samplings, the niche overlap observed in the two environmental variables were higher or significantly higher than expected by chance, suggesting that niche segregation does not exist due to competition within these variables. Similar results were observed for temporal distribution. Some evidence of niche segregation was found in vertical space and horizontal space variables where some samples presented lower overlap than expected by chance. The results pointed out that community's assemblage could be mainly shaped in two ways. The first is that species with determined habitat requirements fit into unoccupied niche spaces. The second is by niche segregation in the vertical space distribution variable.

## Introduction

Many butterflies and other insects fly toward hilltops and mountaintops in order to find a mate, where the efficiency of sexual encounters is increased as the mating area is reduced (Shields 1967; Alcock 1987; Pe'er et al. 2004). This phenomenon, known as “hilltopping”, is somewhat similar to leks established by some birds, beetles and flies and it has been extensively studied by several authors (Shields 1974, 1967; Callaghan 1983; Scott 1983; Ehrlich 1984; Baughman and Murphy 1988; Tyler et al. 1994; Salazar 1996; Pe'er et al. 2004). However, there are few studies that address this phenomenon in a multiple-species context (*e. g*Navez and Ishii 2007).

Hilltops can have a small area relative to the number of species that make use of them, suggesting daily or seasonal activity patterns in taxa occurrence and abundance. Moreover, competitive interactions are important mechanisms that structure natural communities. In other insects, it is widely accepted that competition can shape community assembly to a great extent (Hölldobler and Wilson 1990; Albrecht and Gotelli 2001). Hilltops that are used for mate location by Lepidoptera represent a good ecological study model for several reasons: a) species richness can be very high, b) the abundance of single species can be high, c) the area in which individuals fly is small and d) individuals can be in direct interference when they carry out courtship and territorial activities. Though, hilltopping has been noted in several families of Lepidoptera, the Lycaenidae are often present in higher abundance in neotropical mountains. Prieto and Dahners (2006) have registered more than 80 species of Eumaeini (Lycaenidae) that regularly or sporadically visit San Antonio's hilltop (2.200 m.a.s.l) in the “western cordillera” of the Colombian Andes, and more than 90 Eumaeini species in another hilltop located at 1750 m.a.s.l.

Since a large quantity of resource axes exist that can be distributed among the species, if two species show total overlap in some niche characteristic, it is possible that segregation occurs along another characteristic. Schoener (1974) argues that microhabitat, diet and temporal activity are the three most important distribution axes for a niche and that the largest difference occurs between the first two.

The null model usage for community ecology is summarized by Schoener (1974) who states that niche overlaps observed throughout nature are smaller than those expected by chance. Thus, it can be determined whether the observed segregation is caused by competition or by chance. An analysis with null models compares overlaps in a real community with the average overlap in random communities created for the model.

In this study, the distribution of a Lycaenid assemblage on a hilltop was analyzed on five niche axes. Differences in the usage of these niche axes by the most abundant species were determined and the community overlap was compared with a null model to assess whether the segregation exists as a product of interspecific competition.

## Methods

### Study site

**Figure 1.**
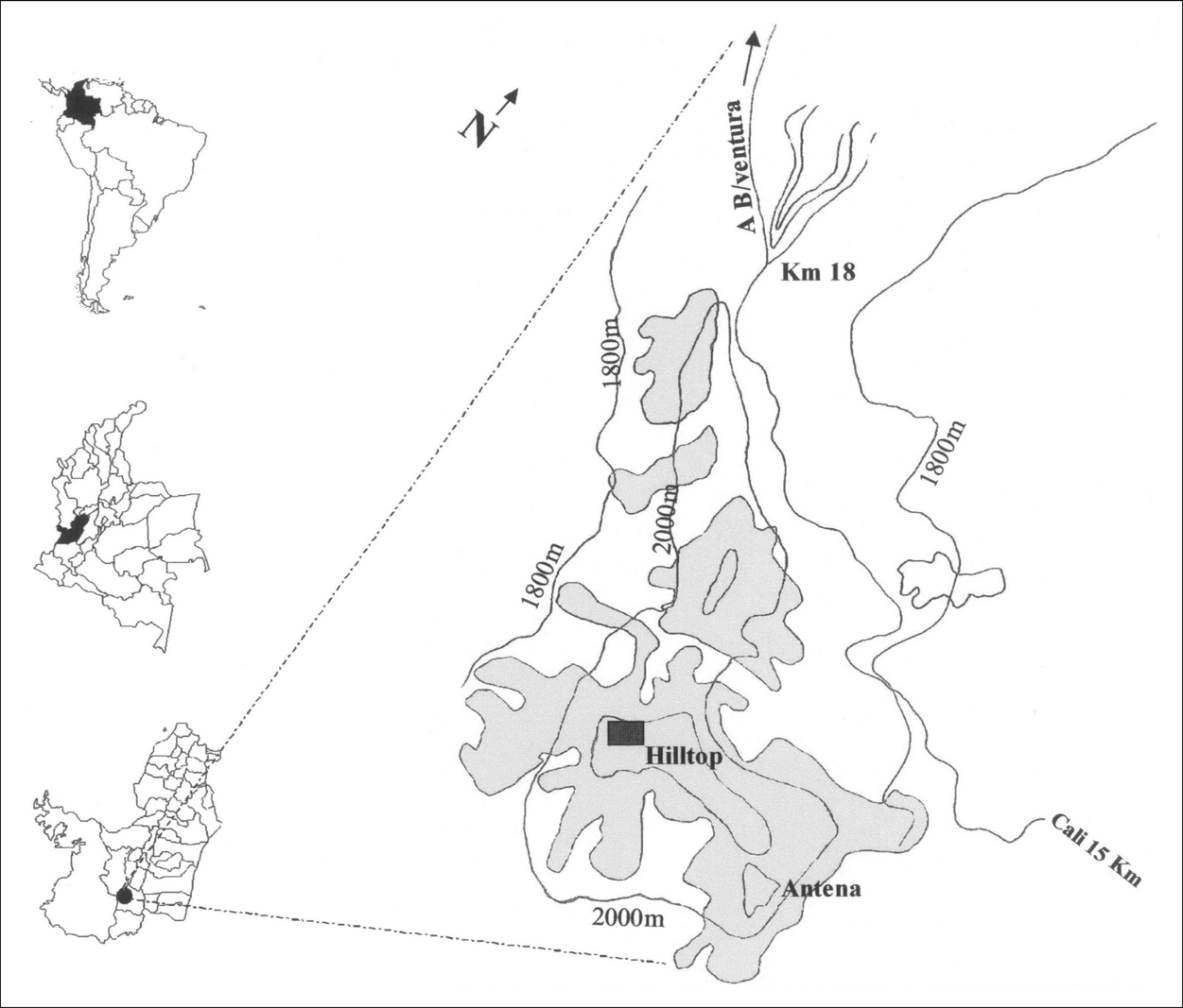
Map of the study area. Rectangle shows the hill studied.

San Antonio hill is located in the “western cordillera” from the department of Valle del Cauca 15 kilometres west of Cali, Colombia, near Buenaventura and in the water line division of the Aguacatal and Dagua river basins (Figure 1). San Antonio is part of a 700 hectares system of forest patches between 1800 m and 2200 m above sea level. Currently, this region is represented by an “archipelago” of forest fragments and countryside houses (Kattan et al. 1994). The San Antonio's forest is classified as a montane humid forest with an annual average temperature of 15°C and an average annual rainfall of 3000 mm (Holdridge 1967).

### Specimen sampling

The specific sampling location was a small sub-hill that constitutes San Antonio's highest location at 2200 m. The hilltop had an area of 200 m2 (12 m × 17 m) and the maximum vegetation height was 7.5 m. A detailed description of the sampling zone and adjoining forests can be found in Bálint et al. (2003), and Prieto and Dahners (2006). Twenty one surveys were carried out by two collectors. Each survey consisted in seven hours of collecting (between 8:00 and 15:00 hours) on days with a cloud cover lower than 40% between June-October of 2004 and June-October of 2005. All individuals that were perching on tree leaves were collected, using a butterfly net of 5 m or 1.80 m in length. The following variables were noted at the moment of capture: perching height over the vegetation, capture place and hour, environmental temperature at 1 m over ground, and light intensity. The environmental temperature was measured with an Onset (www.onsetcomp.com) HOBO-XT HTA data logger. The light intensity was measured with an Onset HOBO-Light HLI data logger and calculated as Log Lum/m^2^.

### Statistical analysis of overlap index and null models

Based on Kruskal-Wallis tests, Dunn multiple comparisons were carried out in order to determine if there were significant differences in use of five variables measured for the 12 most abundant species found at the hilltop. Data of species distribution on the hilltop were used to calculate overlaps in five different niche axes as follows:

- Niche overlap in the environmental temperature. It was determined whether segregation existed among the species in several environmental temperatures. Each row of the data matrix represented a species and each column represented different environmental temperatures between 24 and 40° C in gradations of 2° C. Entries in each cell represented number of individuals captured from each species, perching or flying, in each temperature range.
- Niche overlap in light intensity. For this analysis, each row of the data matrix represented a species and each column represented light intensity ranges of 0.4 log lum/m2 for a total range between 3 and 5 log lum/ m2. The entries in the matrix represented the number of individuals observed in perching or flying state at each range.
- Temporal niche overlap. In this matrix, each row represented one species and the columns represented each hour between 9:00 and 15:00 hrs. The entries in the matrix represented the number of specimens observed during each hour for each species.
- Vertical space niche overlap. For this analysis, each row represented a species and each column a 2m range for 4 total ranges between 0m and 8m. The entries in the matrix were the number of specimens observed in perching behaviour for each height range.
- Horizontal space niche overlap. In this matrix, each row represented a species and each column a perching place. This is a vegetal unit (tree, bush) labelled with a distinctive letter that can be physically differentiated from others. The entries in the matrix represented the number of individuals captured in each perching place.

Niche overlap among each pair of species was calculated with the Czechanowski index (Feinsinger et al. 1981). The range for this index approaches 0 for species that share no resources and approaches 1 for species pairs that have identical patterns of resource utilization. Afterwards, statistical significance of overlap patterns for each variable was calculated utilizing null models in 3 randomization algorithms proposed by Lawlor (1980). These algorithms have been systematically compared by Winemiller and Pianka (1990):

Algorithm RA2: This algorithm creates communities by substitution of the observed resource utilization in each cell of the matrix with a random number between 0 and 1 but retains the 0 position of the original matrix. This algorithm was utilized to analyse the vertical space overlap during the whole sampling period. As well as algorithm RA4, if some species do not utilize a particular category in absence of competition, this structure will be seen reflected in random communities.

Algorithm RA3: This procedure retains the observed utilization of the resource, but randomizes its position inside the matrix. In addition, it modifies the zero states in the original matrix. It was utilized to measure the overlap on horizontal space distribution as well as on environmental temperature and daily distribution.

Algorithm RA4: This procedure retains the observed utilization of the resource and randomizes its position inside the matrix, but retains the zero states in the original matrix. It was utilized to analyze distribution on light intensity and vertical space because, when retaining zero states it maintains real community structure due to biological factors. This means, if all height ranges are not utilized in absence of competition by some of the species, this characteristic will be reflected in random communities.

For each matrix, the utilization data were randomized according to these four algorithms and 1000 null assemblages were created. Subsequently, the average of the overlap index of species pairs from the observed assemblage was calculated and compared with the mean distribution of communities simulated. All the simulations were carried out with the Ecosim700 program (Gotelli and Entsminger 2001).

## Results

### Resource partitioning

**Figure 2.**
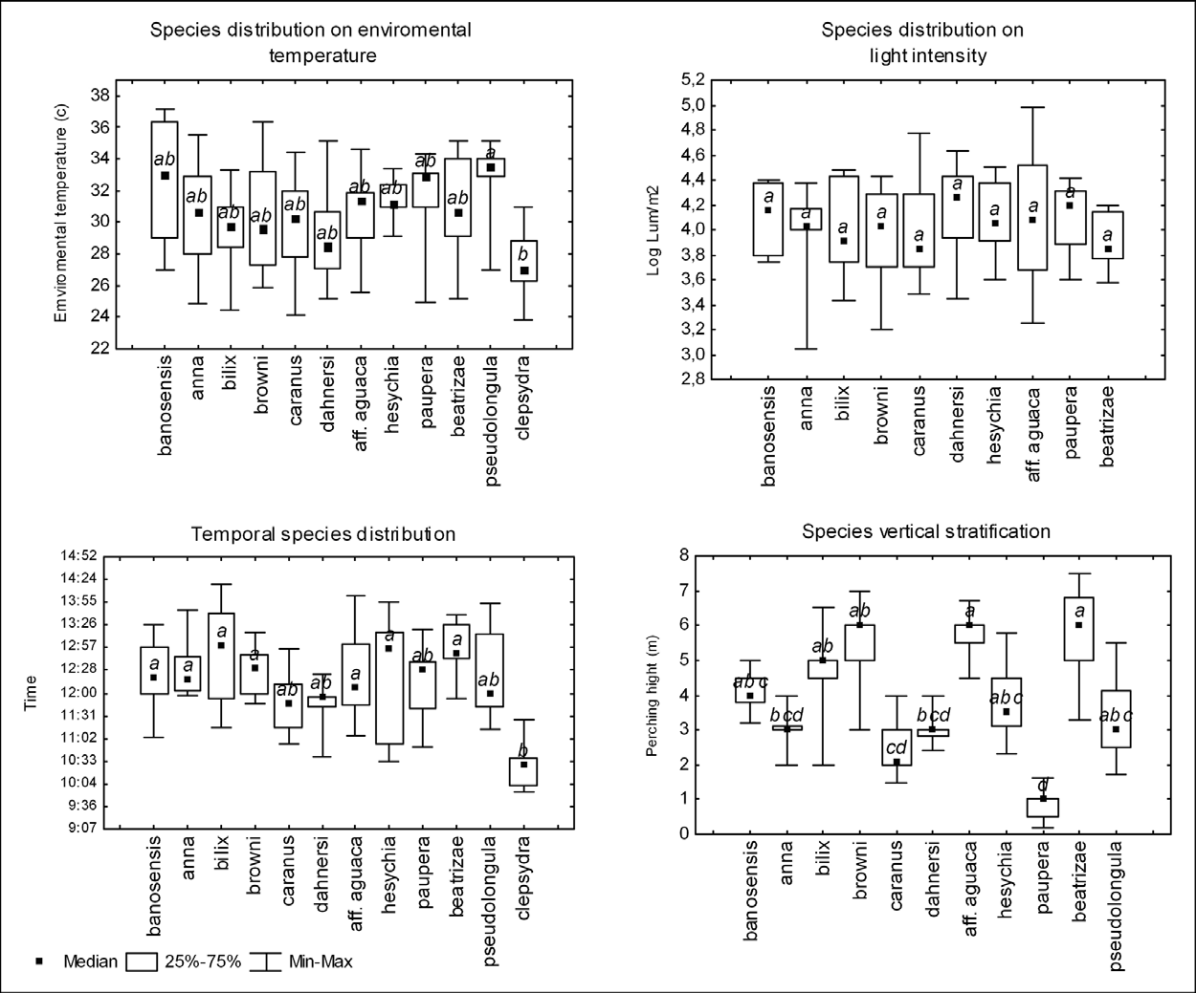
Resource partitioning on four niche axes for the most abundant species in San Antonio hill. Same letter species were not show significant differences in resource use.

In this study 42 Eumaeini species were recorded, but all the analyses were carried out only with the 12 most abundant species (Table 1). Significant differences were found in 4 of the variables used by the butterflies (Figure 2): environmental temperature (Kruskal-Wallis *H* = 20.70, *df* = 11, *p* = 0.03); temporal distribution (Kruskal-Wallis *H* = 38.96, *df* = 11*, p* = 0.0001); space/horizontal distribution (Kruskal-Wallis *H* = 26.17, *df* = 11, *p* = 0.006) and space/vertical distribution (Kruskal-Wallis *H* = 68.07, *df* = 10, *p* = 0.000). There were no significant differences on the light intensity variable (Kruskal-wallis *H* = 8.40, *df* = 9, *p* = 0.49).

**Table 1.**
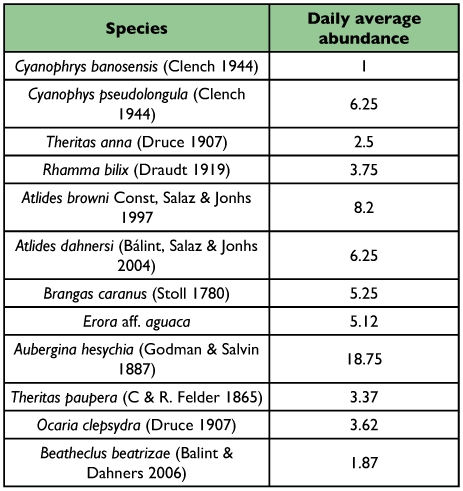
Most abundant species found on San Antonio Hilltop.

### Segregation in environmental variables

**Figure 3.**
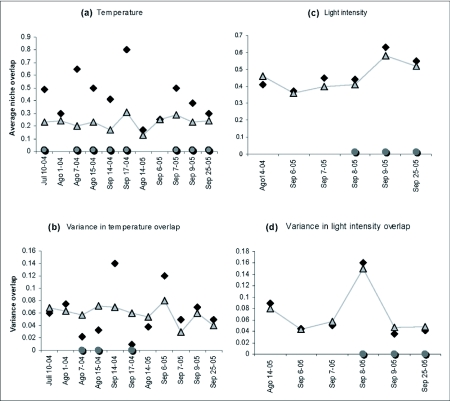
Observed and expected environmental temperature niche overlap (a, b) and light intensity (c, d).

Relatively high overlap indices occurred in the environmental temperature variable. The average overlap observed was significantly higher than the expected value for 8 of the 11 samples (Figure 3a). Results from variances were heterogeneous; 6 samples presented higher observed variance than expected by chance. In August 7th, August 15th and September 17th 2004, the observed variance was significantly lower than expected (Figure 3b). Three of the six samples for the light intensity variable showed a significant higher observed overlap than expected by chance, only the sample from August 14th 2004 showed a lower observed overlap than expected but it was not significant (Figure 3c). Three of the samples showed a higher observed variance than expected; this difference was only significant on 8th September of 2005 (Figure 3d).

### Segregation in spatial variables

**Figure 4.**
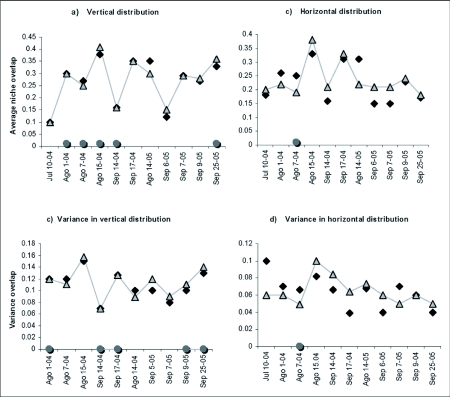
Observed and expected vertical space distribution overlap (a, b) and horizontal space (c, d).

Results for the vertical space distribution variable were heterogeneous. However, low overlap indices were present (<0.5). Seven of 11 observed overlap samples were higher than expected and the differences were significant in three of these samples. Four samples presented lower overlap than expected, on August 15th 2004 and September 25th 2005 these differences were significant (Figure 4a). Variance of five of eleven samples was higher than expected; three of these samples were significantly higher. The observed variance of September 9th and September 25th of 2005 were significantly lower than expected (Figure 4b).

Most of the samples (8) for the horizontal space distribution variable showed lower overlap than expected and none of these samples were statistically significant. The observed overlap index in August 7th 2004 was significantly higher than expected (Figure 4c). In 5 samples, the variance was higher than expected; but none of these were statistically significant. The observed variance from August 7th 2004 was significantly higher than expected (Figure 4d).

### Segregation in the temporal variable

**Figure 5.**
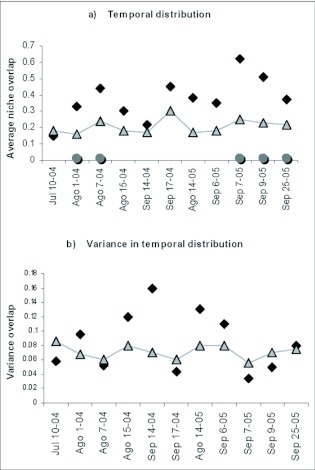
Observed and expected temporal niche overlap (a); variance in niche overlap (b).

Results for the temporal variable were very homogeneous. Overlap observed for the temporary variable (hours/day) was always above the expected (Figure 5a). Significant differences were found among two samples from August as well as 3 samples carried out in September. The overlap variance was more heterogeneous. No significant differences were found for this variable (Figure 5b).

**Figure 6.**
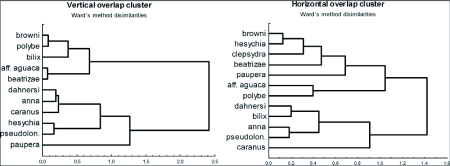
Ward's method dissimilarities cluster for spatial variables. Species near to left axis was overlapped. *Atlides polybe* is included.

A dissimilarity analysis showed three groups of species that perched at three different heights above the vegetation. The species *Atlides browni, Atlides polybe, Rhamma bilix, Erora aff. aguaca* and *Beatheclus beatnzae* perched together above 4 meters. *Thentas anna, Brangas caranus* and *Atlides dahnersi* perched together at heights between 2 and 4 meters. *Aubergina hesychia and Cyanophrys pseudolongula* showed a wide range of overlapping perching heights (Figure 6). *Thentas paupera* was separated in an external group because it is the only species that exclusively flew or perched under 1.50 m. The dissimilarity cluster for the horizontal space variable was totally different from the vertical space variable. As a result, when two species shared the same tree (horizontal distribution) they were segregated at different levels on the tree (vertical distribution), which may be a strategy to avoid territorial fighting (Figure 6).

In an unrestricted analysis carried out for all the variables, only vertical space segregation showed lower overlap than expected by chance (RA2); observed variances were significantly higher than those expected (Table 2). The overlap found in other four variables was significantly greater than expected. The horizontal space distribution showed a higher variance than expected (Table 2).

## Discussion

Significant differences in temperature during the activity period were only found in *Ocaria clepsydra* whose flight activity was between 23–30° C. Significant differences were not found in the temperature and light intensity variables. This result may be common to many insect assembles and ectothermic animals. Environmental factors such as temperature and light intensity are related to community and population ecology by thermoregulation and flight capability (Kingsolver 1985). Detailed studies have shown that diurnal butterflies require thorax temperatures between 33 and 38° C (Kingsolver 1985). Since the thorax temperature depends of the heat exchange with the environment, insects have developed a set of behavioural mechanisms that assures an optimal flight temperature. Certain behaviours such as oviposition and courtship only occur when particular optimal flight temperatures are reached (Roland 1982; Kingsolver 1985; Hirota and Obara 2000).

The body temperature for smaller sized butterflies such as the Eumaeini can change drastically within 30–60 seconds after an environmental change (Kingsolver 1985). Therefore, the observed large overlap in the response of species to environmental temperature is because all of them require an optimal temperature between 30–38° C when searching for a mate (Figures 2 and 3a). Thus, it would be difficult to develop niche segregation by competition or differences in resource utilization when physiological constrictions force a great number of species to be active in the same temperature range.

Light intensity overlap was higher than for environmental temperature. Since endothermic heat generation by wing movement before flight or regulation of hemolymph circulation are not common mechanisms of diurnal thermoregulation in butterflies, the main mechanism by which they regulate heat gain is through body position and orientation in relation to the sun. Light intensity is probably more important for thermoregulation than environmental temperature in this group because these butterflies can elevate their body temperature in the presence of high solar radiation even when environmental temperatures are low. In the premontane Andean zones, cloud movements are regulated by turbulent air flow which tends to be chaotic and unpredictable, and, as a result, the amount of solar radiation that reaches the vegetation varies. Therefore, when there is relatively higher solar radiation lycaenids carry out mating activities, but if cloud cover reduces the solar radiation, then mating activity is reduced. *O. clepsydra* probably possesses more efficient mechanisms for thermoregulation conferring the capacity to make better use of the time of day when temperature is low and space competition is less aggressive.

Significant differences were found in three other variables: vertical space distribution, horizontal space distribution and temporary distribution. The variable that presented higher utilization differences by the species was the vertical space variable (Figure 2). Differences in the vertical distribution of butterflies have been found in various groups of tropical butterflies, mainly in Ithomiinae (Beccaloni 1997; DeVries et al. 1999b) and various groups of Nymphalidae (Fermon et. al 2005; DeVries 1988; DeVries and Walla 2001; DeVries et al. 1997; DeVries et al. 1999a). These studies have concluded that stratification is caused because females tend to fly at the same height where the host plants thrive, thus increasing interaction opportunities. In similar ways, males increase the opportunities for interaction when they fly at the same level as the females (Beccaloni 1997). Although it is possible that for some Eumaeini species perching stratification is a consequence of host plant distribution, observed stratification in hilltopping Eumaeini is more constrained than in other butterfly groups. Within an 8 meter range, 3 or 4 perching and flight strata can be found in this group. Although host plants for Eumaeini are largely undocumented, it is unlikely that these plants have a particular distribution in a vertical space. For example, the species *A. browni* and *A. dahnersi* showed significant differences in their perching heights. It is known that larval states from *Atlides* species feed on Lorantaceae (Valencia et al. 2005). These plants are parasites on other trees and their germination on host plant branches occur after the seed passes through the digestive tract of birds which have fed on the plants, which later are excreted randomly on the guest tree branches. Thus, there is no vertical stratification of Loranthaceae that could produce stratification of perching in these two *Atlides* species.

**Table 2.**
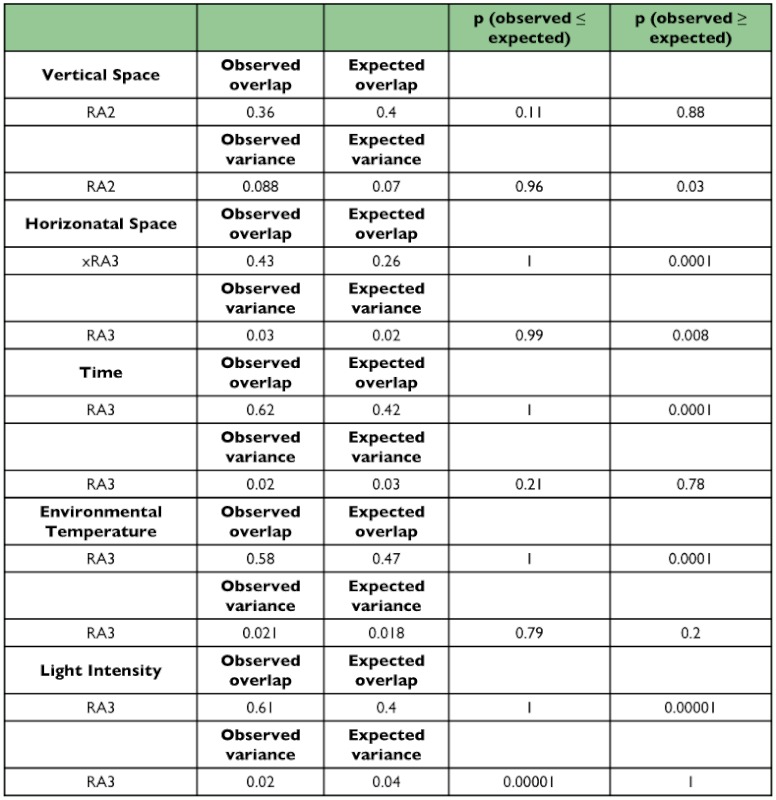
Unrestricted analysis for the five studied variables.

Various factors might generate vertical stratification in butterflies; micro-climate differences such as wind, temperature, and light intensity (DeVries 1988; DeVries et al. 1997) or structural variations in vegetation and pressure by predators (Hill et al. 2001; Schulze et al. 2001; Fermon et al. 2005). It is probable that small differences in light intensity use, as observed indirectly in *O. clepsydra,* allow this type of stratification in Eumaeini. These differences may allow temporal- space segregation when interspecific competition is also present.

Several authors have reported temporal-space separation in butterfly species that use the same mating locations (Callaghan 1983; Brown and Alcock 1990; Hafernik 1982; Turner 1990; Rutowski 1991). Callaghan (1983) has considered temporal-space separation as a prezygotic isolation mechanism of Riodinidae. He found that sympatric species belonging to the same genus were isolated because copulation is carried out in different topographical places at different times. His results agree with those found in this study. The Eumaeini display adaptive perching behaviour as illustrated by differences in perching places at different times. Consequently, these butterflies may save time and energy by avoiding interactions with other species through this difference in the location of perching.

The way in which time regulates ecological interactions and shapes ecological communities has been poorly studied (Jaksic 1982; Schoener 1986; Kronfeld-Schor and Dayan 2003). This theory postulates that time distribution promotes coexistence among competitive species (Schoener 1974; Richards 2002; Kronfeld-Schor and Dayan 2003). Dann (1981) suggests that different activity patterns require different evolutionary adaptations and therefore, close phylogenetic species are usually active during the same daily cycle section. This hypothesis implies that species are evolutionarily constrained in their patterns of activity and their flexibility to adapt to the environment, as suggested by Schoener (1974), is limited (Kronfeld-Schor and Dayan 2003).

Although significant differences were found in the schedule of activity, these differences were linked to only to few species in the community and they were directly related to environmental temperature and light intensity. Therefore, species such as *O. clepsydra* and its capacity for flight in low environmental temperatures and high light intensity values, allow it to be active earlier in the morning when temperatures are comparatively lower and light intensity comparatively higher.

It is not enough to show that species differ in resource use in order to argue that there was a reduction in niche overlap, because species can differ in resource utilization in the absence of competition (Connell 1980; Gotelli and Entsminger 2001). Analyses with null models illustrate conclusively that segregation due to competition did not exist for light intensity and environmental temperature since the majority of samples exhibited overlap indices higher than expected (Figure 3). Evidence of segregation due to competition can be found in spatial variables (Figure 4).

Vertical space niche segregation due to competition is consistent with the fact that overlaps on 15th August 2004 and 25th September 2005 were significantly lower than expected. In addition, the unrestricted analysis for this variable showed a lower overlap than expected (Table 2). Eight of eleven samples for horizontal space segregation had lower observed overlap than expected (Figure 4c, d). On this particular site, there was no niche segregation in the hour of activity variable (Figure 5). It is possible that spatial variables showed segregation more frequently due to competition on hilltops, since they are not restricted by physiological constrictions for use and can be exploited by the species when a small adaptive advantage in segregation exists.

Taking into account the factors for resource usage, and the small evidence for segregation due to competition in this site, it is probable that the community assembly at the hilltop peak could be shaped by two paths: 1) Species arriving to the hilltop can be part of the hilltop community because its biology easily adjusts to an available niche space. This could be the case of two *Theritas* species that flies beneath one meter (Prieto 2006). *T. paupera* can form part of a hilltop community if it does not find competitors occupying its favored perch height. It has been observed that *T. mavors* and *T. paupera* never share the same hilltops in the Aguacatal river basin. Moreover, in some cases it has been observed that species shift, but are rarely observed flying together (Prieto 2006). Certainly, if individuals of these species meet together on a hilltop, there will be some competition. However, probably the habitat requirements of these species are stronger than space-temporal segregation ability. If so, competition could force one of the species to leave the hilltop.

2) Another reason that might be influencing the structuring of a hilltop community is interspecific competition at least in the vertical space variable. It is very probable that species such as *A. browni* and *A. dahnersi* are segregated in vertical space by competition. On San Antonio hill, *Atlides* species generally fly in the canopy during midday hours. Although *A. browni* maintains this behaviour, *A. dahnersi* uses the vegetation portion under the canopy and rarely perches directly over it. In addition, it prefers sunny hours or before midday. Because these two species are very similar, phylogenetically related and abundant, segregation in a niche axe would save a large quantity of energy during recognition flights to the other species when there is a mating search.

## Conclusions

Differences existed in hilltop use for space and temporal variables. These differences could be based on diverse physiological mechanisms or behaviours that allow the species to utilize one or another niche category. Nevertheless, other niche factors such as environmental conditions are intimately connected with the physiological constrictions and do not provide a wide action range which could permit differences in resource use.

Vertical stratification has been explained satisfactorily in other butterfly groups and in other habitats. For mate location and for diverse groups such as Lycaenidae, it is very probable that these explanations are not satisfactory. Several species in a small area of such importance for their life cycle makes competition more energetic and direct. This creates a variety of adaptive strategies that can shape a community's structure along the hilltops. The hilltop represents a natural laboratory to study competitive interactions in a community level. Nevertheless, conditions in such places allow adaptive mechanisms different from those already found in conventional habitats.

Our study is an initial attempt to assess the hill topping behaviour in the context of community structure and provides a general view and basic information about how tropical Andean Eumaeini species use this resource. However, our study only focused on one hilltop and on distributional comparison of the same taxonomic group. It is necessary to make comparisons among different peaks to clarify whether resource repartition found in this study is a unique or a generalized phenomenon. Since competition between species can also be expressed in the absence of species when certain other species occur, further studies using methodologies such as mark-release-recapture will be useful to analyse if the set of species observed is dependent on other species.
